# Topography, Climate, and Diseases of Washington Territory

**Published:** 1872-07-15

**Authors:** F. R. Payne

**Affiliations:** Marshall, Ill.


### Original Communications.

### TOPOGRAPHY, CLIMATE, AND DISEASES OF WASHINGTON TERRITORY.

*Read before the Clark Co. Medical Society, July 3d, 1872.*

BY F. R. PAYNE, M.D., MARSHALL, ILL.

[*Published by Order of Society.*]

During the last year we visited and spent several months in Washington Territory, and formed the acquaintance of all of the leading physicians of that far-off land.

The western portion of that Territory is generally covered with a dense and very large growth of fir and cedar trees; occasionally we found a small sandy prairie, almost worthless for farming purposes. The fir and cedar is from one to three hundred feet high and six inches up to eight feet in diameter. This tremendous timber grows upon the sand and gravel hills and spurs of mountains. There are many rivers, but the bottoms of these streams are only from one fourth to one mile in width; rich alluvial and peat soil, upon which cedar grows luxuriantly and in mammoth proportions; the fir seems to flourish best on the sandy and gravel ridges. The bottoms of the rivers are subject to overflow, but very little injury is done to the farming lands by these annual inundations. Miasmatic diseases are unknown in that country; the summers being cool, and hill lands principally sand and gravel, miasmatic poison is not generated. The capital, Olympia, is a flourishing little city of about eighteen hundred inhabitants, situated at the head of Puget Sound, one hundred and eighty-five miles from the Pacific Ocean by water, but on a straight line westward by land, not over seventy-five miles. This dense forest between the cascade and coast ranges of mountains is about three hundred miles long and one hundred and twenty-five wide—is called Western Washington. A large proportion of this territory is worthless for agricultural purposes, but we find many rich strips of black alluvial and peat soil, from two feet to ten in depth, in the river bottoms and low grounds between the hills. There are also cranberry swamps, many of which are easily drained, all of which are very fertile. In all of these low grounds the water is supplied by mountain springs, consequently, no poisonous elements are produced by decomposition of vegetable matter.

The climate of this region during the summer and fall is delightful; no winds or excessive heat; venomous reptiles and insects are unknown; the days are pleasant and nights quite cool, sufficiently so to prevent the growth and prosperity of musquitoes and frogs, the stings and music of which are not felt or heard in that far-off land. Puget Sound is abundantly supplied with all kinds of salt water fish, oysters, clams and crabs, which furnish cheap food for the inhabitants, and especially Indian tribes. Puget Sound proper, which extends from Seattle to Olympia, a distance of fifty miles, is the finest harbor perhaps in the world. It is from one to two and a half miles wide, with ample water for the largest class of ships and steamers, and no rocks or hidden obstructions. The mariner feels perfectly safe when his vessel reaches this magnificent harbor.

The summers are so invigorating and lovely, that during that portion of the year there is but little sickness, but the winters are very wet. The annual rain fall generally begins the first of November and continues with slight interruptions until about the first of May. Although in forty-seven and eight degrees north latitude, there is very little cold weather, yet from our own observations and what we could learn from the best physicians in that country, during the winter and spring months they have a great deal of sickness. The wet weather and damp atmosphere during the winter and spring is very productive of rheumatic fevers, and in that region there is no thunder and lightning, consequently very little if any electricity. This fact doubtless explains the frequent occurrence of rheumatic forms of neuralgia and nervous diseases generally. We witnessed many cases of partial paralysis, lumbago, spinal irritation and other forms of nervous disorder. There are a great many cases of amenorrhœa and emansio-mensium, a large majority of which result fatally. More than half the young girls die from suppression and retention of the catamenial flow, and the consequences resulting from this abnormal condition of the system. Drs. Rufus Willard and A. H. Steele, intelligent physicians, who have resided in that country for many years, and nearly all the time at Olympia the capital, were very communicative and gave me much reliable information in regard to the diseases of that territory. Many of the inhabitants are a mixture of the Anglo-Saxon and Indian, and a large majority of the half-breed girls die from emansio-mensium and its consequences. They are full of life and vigor until the age of puberty ; then gradually pine away and die. Some never menstruate, while others have a slight flow, and then disease of the lungs, and low forms of fever supervene, causing the systemic waste to be much greater than the supply, and great emaciation, and death is the result. In the first settlement of the north-west, the Hudson Bay Company formed intimate commercial, and to some extent social relations with the Indian tribes of Oregon, Washington Territory and Vancouver's Island. Many white men married squaws and now have large families, and have by prudence and industry become quite wealthy, and are now educating their children ; but all careful observers are fully satisfied that this mixture of races results in an unhealthy and short-lived offspring. We had the pleasure of visiting a number of Indian reservations, and on the Skokomish river, near the head of Hood's canal, we spent several weeks. Mr. Eells, a congregationalist, and very worthy man, is superintendent, and the physician in charge is Dr. W. Price. At this point they have nine or ten hundred Indians, and Dr. Price is a conscientious man who is trying to relieve them from the terrible diseases which have been introduced among them by vile and unprincipled white men. A large proportion of this tribe (and we learn that the same is true with all,) are suffering with the worst and most loathsome forms of syphilitic disease. We will present you with the treatment of Dr. Price, who has had many years of experience in managing this disease on the frontier, and among these degraded people. He first prepares the system and then gives them freely the *Liquor Arsenici et Hydragyri Iodidi.* This seems to have a very happy effect, and to use the language of Dr. Price, "there is no remedy in the materia medica that will have such universal good effects in this loathsome disease." He informed me that he had used it in hundreds of cases and was delighted at the good results. In many of these Indian tribes the entire system is saturated with syphilitic poison. They have it in all forms, primary, constitutional and tertiary, and in the latter form many of them present a horrid appearance ; ulcerated throats, dark blotches, pains in the bones, nodes, buboes and exceedingly offensive soft chancres. Many of the tribes on the Pacific slope are rapidly dying out from the effects of this and other contagious diseases. Their nomadic lives and filthy mode of living renders it almost impossible to secure the beneficial effects of remedial agents.

It may interest many of the members of this society for me to briefly refer to the historical origin and spread of syphilitic diseases. Its origin is lost in the remotest antiquity, and its early investigations were instituted from religious motives and pronounced a punishment inflicted by God for the extraordinary sexual transgressions of mankind. Still later it was viewed from a political stand point, and was used to gratify national animosities. The French called it "*Mal de Naples*," the Italians "*Mal Francese*," and the Germans "*Franzosen.*" The Spanish charged the American Indians for inflicting their people with this loathsome disease, and plead this as justification for their cruel and inhuman treatment of that ignorant and degraded race.

The history of syphilis is divided into three periods—the first extending to the close of the fifteenth century, the second to the time when Fernel discovered that it was propagated by a specific poison, and the third period extends to the present day.

In the books of Moses we find allusions to contagious discharges from the urethra. It is contended by many able men that true syphilitic poison was transmitted to man from the brute creation, like glanders and small-pox. We find similar allusions in the writings of ancient Greek physicians. Hippocrates speaks of ulcers of the sexual organs, pustules of the penis, and many other diseases of the sexual organs which clearly indicate syphilis. Celsus, Galen and other ancient writers describe every variety of chancre and other forms of disease which was communicated to man by unclean females. In 1556, Fernel clearly demonstrated that syphilitic diseases originated in a specific poison, and was strictly a contagious disease. He deserves the credit of eradicating from the minds of thousands the absurd belief in astrological, cosmic or teleological ideas.

In looking over the history, origin and progress of this terrible malady, and what we have witnessed during the past year, the aborigines of America have suffered more with its ravages than any other people on earth. It is no stretch of imagination for us to predict that without a higher degree of Christian civilization, the day is not far distant when the original inhabitants of this continent will become extinct.

We formed the acquaintance of the Superintendent of the Insane Asylum which is now permanently located at the town of Steilacoom. He is a competent and worthy man. There were sixty-seven patients in the Asylum, which is very large for the number of inhabitants. The cause of this vast amount of insanity is not well understood.

From our own observations and what we could learn from the physicians of that country, we are of the opinion that the inhabitants suffer more with diseases of the kidneys than they do in milder climates. The cause of this is that the summers are quite cool—frost almost every month of the year—consequently, there is but very little visible perspiration, forcing upon the kidneys double duty.

We have presented a brief account of the topography, diseases and climate of Washington Territory. There is a great deal that might be said in regard to that country, but we have not time to enter into details. At some future day we will give some account of Oregon, California, their diseases, medical schools, and other matters which may interest the profession.

